# Operator Independent Focused High Frequency ISM Band for Fat Reduction: Porcine Model

**DOI:** 10.1002/lsm.22134

**Published:** 2013-04-26

**Authors:** Robert Weiss, Margaret Weiss, Karen Beasley, Jan Vrba, Jan Bernardy

**Affiliations:** 1 MD Laser, Skin & Vein InstituteBaltimore, Maryland; 2 Czech Technical UniversityPrague, Czech Republic

**Keywords:** adipocyte, obesity, radiofrequency, body contouring, fat reduction

## Abstract

**Background:**

Selective fat reduction has been clearly shown for various methods and energy modalities including cryolipolysis and high intensity focused thermal ultrasound. Mathematical modeling of focused high frequency of the EM spectrum has indicated that selective heating of fat is possible using wavelengths not previous explored. The purpose of this study was to demonstrate in the porcine model that selective heating of fat is possible with a non-contact, operator independent device.

**Methods:**

High frequencies of the Industrial, Scientific and Medical (ISM) RF band were utilized to reduce abdominal fat in a porcine model. Practical application of mathematical modeling allowed an auto-feedback loop to be developed to allow operator independent adjustment of energy to maintain subcutaneous fat at 45–46°C while overlying skin remained at 40–41°C.

**Results:**

Treatments of three Vietnamese pigs were performed under anesthesia in a certified veterinary facility. Gross and microscopic histologic results demonstrated a marked reduction in adipocytes of the treated area after 4 treatments of a total of 30 minutes each, with incremental fat diminution after each treatment. A final 70% reduction of the abdominal fat layer was seen in the treated areas. Duplex ultrasound revealed a reduction of fat layer from 7.6 to 2.9 mm. Histologic evaluation revealed that epidermis, dermis, and adnexal structures such as hair follicles were unaffected by the treatment, while adipocytes were significantly affected.

**Conclusion:**

A new model of fat reduction using high frequency RF has been successfully achieved in a porcine model. This has very positive implications in the development of an operator independent, contact free device for reduction of fat in clinical practice. Lasers Surg. Med. © 2013 Wiley Periodicals, Inc.

## INTRODUCTION

Multiple modalities to induce adipocyte apoptosis in order to reduce pockets of fat non-invasively have recently become available. These modalities primarily aim at targeting the properties of fat which differentiate it from skin and muscle, thus resulting in selective removal or dissolution of fat otherwise known as lipolysis. Currently available non-invasive fat removal methods utilize heating or cooling utilizing, laser, radiofrequency, and ultrasound sources to more selectively target adipocytes 1–5.

The medical use of RF is based on an oscillating electrical current forcing collisions between charged molecules and ions which are then transformed into heat. A dielectric, such as fat, is an insulator with the ability of inner polarization. Adipose tissue contains electrical dipoles. The direction of dipoles is chaotic and polarization arranges dipoles in one direction. Dielectric polarization requires that every electrical dipole is rotated against the polarization of the electrical field. With a rapidly alternating electromagnetic field, all electrical dipoles oscillate. This oscillating movement of dipoles leads to heating up of dipoles of fatty tissue, a principle mechanism of action of high frequency on fat.

This study was designed to investigate an operator independent focused field system designed for contactless deep tissue thermal energy application. The applicator–generator circuitry is engineered to selectively deliver the energy to tissue layer with specific impedance, in this case the adipose tissue layer. This high frequency system focuses energy specifically into the adipose tissue layer, while limiting the delivery to the dermis, epidermis and muscles. A multipolar broad field applicator shapes the electro-magnetic field to optimize the penetration and maximize the treatment area. Using a patented Energy Flow Control™ (EFC™) system this device automatically tunes the tissue-applicator–generator circuitry to selectively deliver the energy to the adipose tissue layer while minimizing the risk of overheating of the skin, muscles, or internal organs as is detailed below.

## METHODS

This study was approved by the Institutional Animal Care and Use Committee (IACUC) and the Committee for Animal Protection of the Ministry of Agriculture of the Czech Republic. Procedures used conformed to accepted practices and to minimize or avoid causing pain, distress, or discomfort to the animals. In those circumstances in which study procedures were likely to cause more than momentary or slight pain or distress, the animals received analgesics or anesthetics as per the Institutional Animal Care and Use Committee (IACUC) at University of Veterinary and Pharmaceutical Sciences Brno.

Four square shaped areas of the skin in *rectus abdominalis* (each square 10 cm × 10 cm) were selected and marked as A, B, C, and D. Hairs were removed from the application area by clipping. The device used to perform treatment was the high frequency field RF device sold as Vanquish™, BTL Aesthetics, Prague, CR. Treatment was performed with the RF applicator placed approximately 1 cm above the skin. Total exposure time (30 minutes) of each treatment was divided into two 15 minute halves. Skin surface was kept in the temperature range from 39 to 42°C during the treatment period.

Animals were under the total anesthesia and under the supervision of the veterinarian during each treatment and during the biopsy. Supervising veterinarians selected the type and the dose of the anesthesia.

All animals were observed for clinical signs, morbidity or mortality once a day during the treatment period. Onset, duration and severity of any signs were recorded. Clinical observations included: (1) skin, eyes, and mucous membranes changes, (2) respiratory, circulatory, autonomic, and the central nervous system, (3) somatomotor activity and behavior pattern, changes in gait, posture and response to handling, presence of clonic or tonic movements and stereotypes.

Blood samples for clinical chemistry were collected from all animals before each treatment, after each treatment and 3 months after the last treatment (recovery period). Blood samples were collected into Tapval tubes without anticoagulation (clinical chemistry), serum samples were obtained by centrifugation at 3,000 rpm for 15 minutes. Serum for clinical chemistry (∼1 ml) was transferred into appropriately labeled and sealed Eppendorf tube and frozen at −20°C or below until transport for analysis.

The treated area was examined before 1st, 3rd, 4th treatment and at the end of the study period by Duplex ultrasound. All animals were individually weighed before each treatment and after the study period. Temperatures of the epidermis and the adipose tissue temperature were measured by thermocouple before treatment, after 5, 12, 15, 20, 27, and 30 minutes of the treatment and immediately 30 minutes of exposure time. Epidermal temperature was monitored with an infrared camera during each treatment. Biopsy samples of the skin and the fat tissue were taken with biopsy needle before treatment, after the first treatment, after 2nd, 4th treatment and 3 months after the last treatment (recovery period). Samples of liver, lung, and skin from all three swine for histopathology exam were taken at the conclusion of the treatment protocol.

## RESULTS

**Fig. 1:**
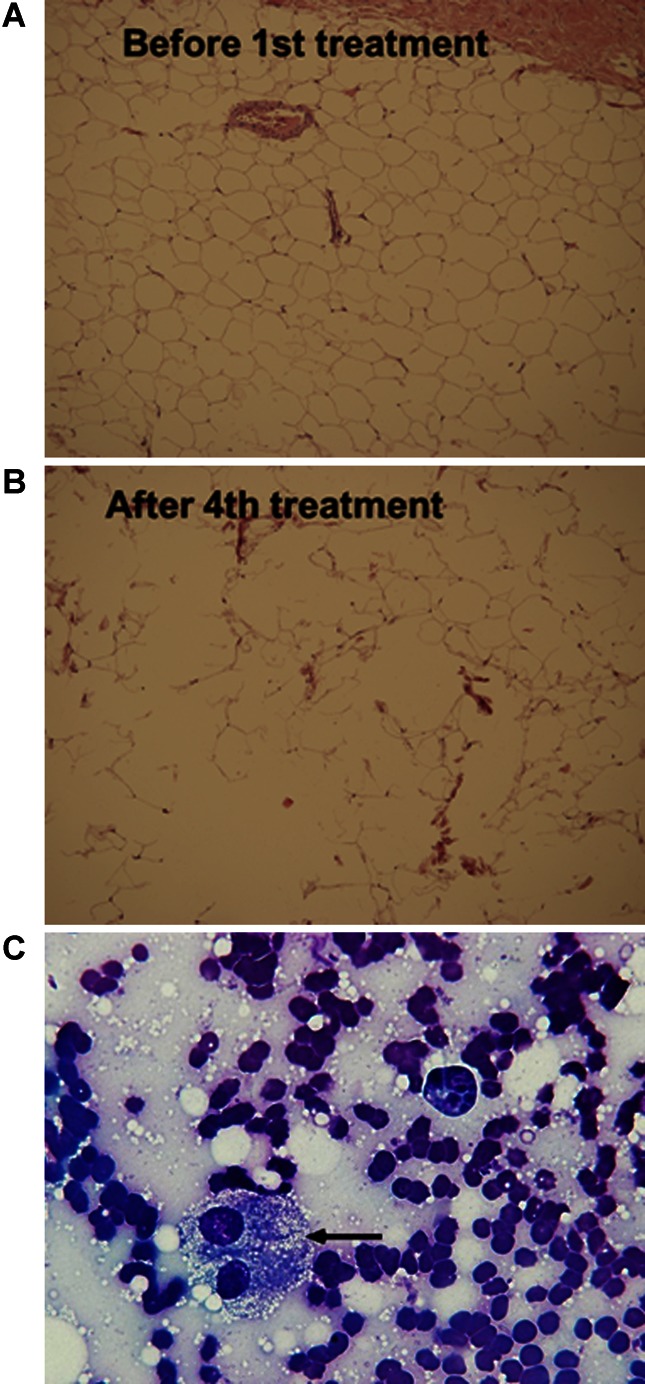
**A**: Normal fat before treatment. **B**: Disrupted fat after 4th treatment. **C**: Foamy macrophages following treatment.

Skin erythema was observed during treatment, usually several minutes after the start of the treatment. Skin erythema resolved within minutes following the cessation of treatment. The first biopsy revealed infiltration of foamy macrophages and neutrophil granulocytes (Fig. 1). Biopsies taken 2 weeks after the third treatment were without signs of panniculitis. Histological analysis of skin biopsy samples taken after first treatment revealed desquamation of superficial layers of epidermis, perivascular infiltration, fragmentation of superficial collagen fibrils, and alteration of the adipose tissue. Significant adipose tissue destruction was found (Fig. 1).

Following histological examinations after the second and fourth treatment showed similar signs as observed after the first treatment with desquamation of superficial layers of epidermis, perivascular infiltration, fragmentation of superficial collagen fibrils, and alteration of the adipose tissue with the exception of sporadic coagulo-necrotic lesions of the adipose tissue. After the fourth treatment the histological evaluation showed adipose tissue destruction in dermal layers. In one subject more frequent fibrotic septa were found after the fourth treatment.

Histological examination of the skin biopsy after the end of the recovery period revealed in all animals local desquamation of epidermal surface, sporadic perivascular infiltration in dermis, focal disintegration in adipose tissue, and thick fibrotic septa.

Histology of the skin samples from dermal depression showed intense desquamation of epidermis, focal mononuclear infiltration of dermal vessels, fragmentation of collagen fibrils, multicystic foci with inflammatory cell infiltration. Adipose tissue was observed to be disrupted along with focal fibrosis.

**Fig. 2:**
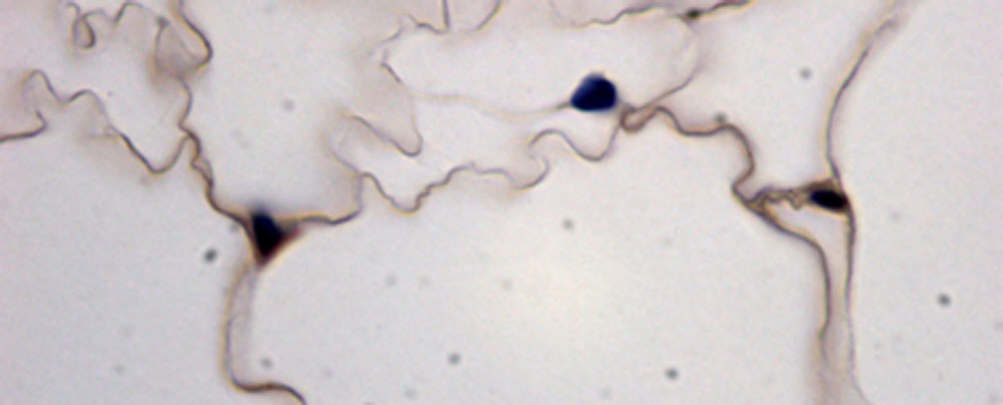
Evidence of apoptosis by TUNEL staining method.

The TUNEL method was used to detect apoptotic nuclei in the samples. Terminal deoxynucleotidyl transferase dUTP nick end labeling (TUNEL) is a method for detecting DNA fragmentation by labeling the terminal end of nucleic acids. TUNEL is a common method for detecting DNA fragmentation that results from apoptotic signaling cascades. The assay relies on the presence of nicks in the DNA which can be identified by terminal deoxynucleotidyl transferase or TdT. It may also label cells that have suffered severe DNA damage. The detected apoptotic index was 13/100 prior to the initial treatment and 52/100 following the last treatment. Evidence of apoptosis is shown in Figure 2.

Serum levels of glucose, bilirubin, urea, ALT, total cholesterol, HDL and LDL cholesterols and triglycerides were unaffected by treatment. Aspartate aminotransferase (AST) serum activity increase was observed in one subject at one time point but was judged not related to treatment.

A final 70% reduction of the abdominal fat layer was seen in the treated areas. Average adipose layer thickness reduction was 6.9 mm. Duplex ultrasound revealed a reduction of fat layer from 7.6 to 2.9 mm. No significant changes in body weight were measured as individual body weights of the animals were within normal range, corresponding to the age and baseline weight.

**Fig. 3:**
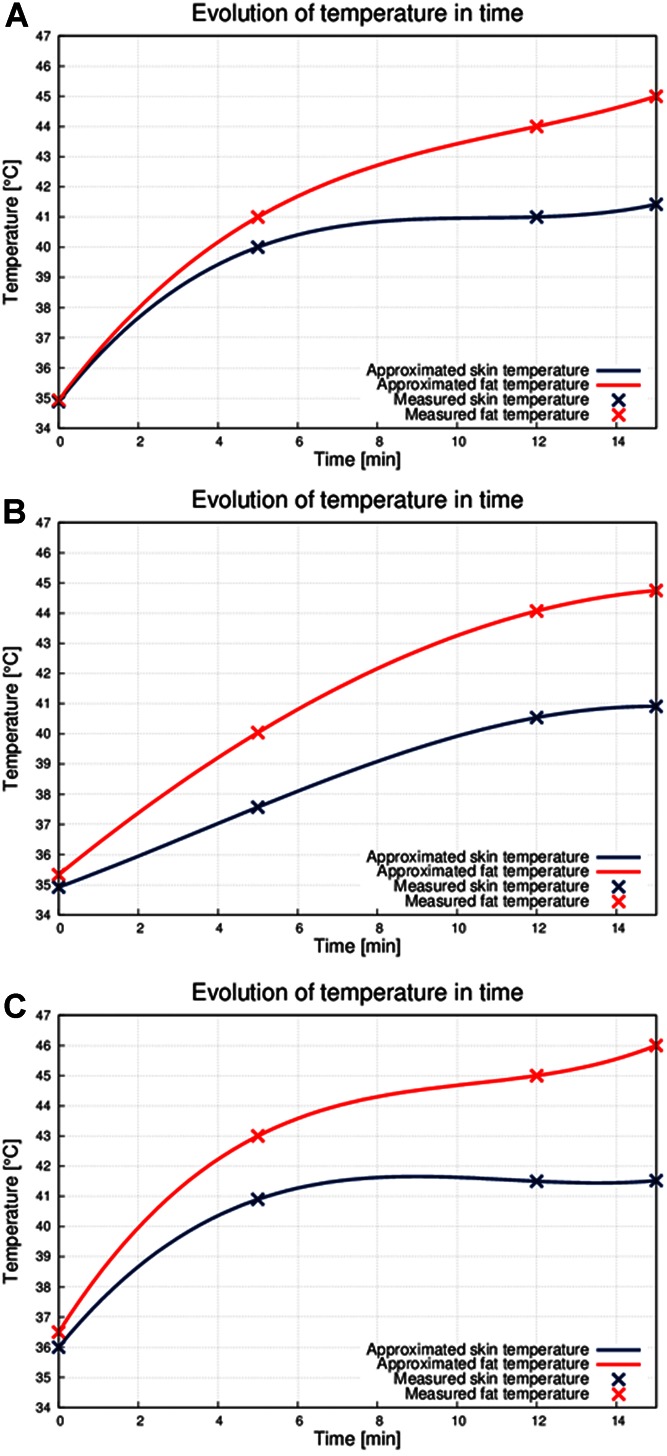
Evolution of temperature over time from 0 to 15 minutes. **A**: Subject 1. **B**: Subject 2. **C**: Subject 3.

**Fig. 4:**
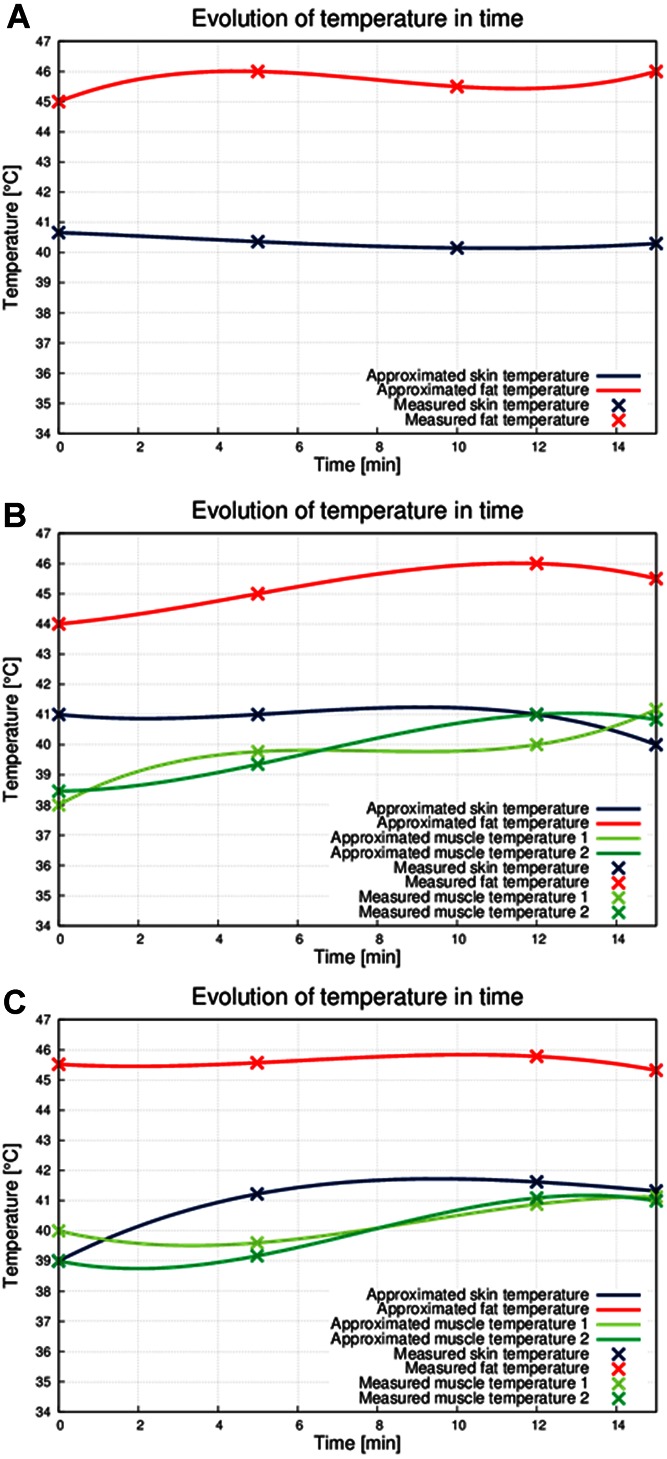
Evolution of temperature over time from 15 to 30 minutes. **A**: Subject 1. **B**: Subject 2. **C**: Subject 3.

The skin temperature and the adipose tissue temperature measurement results were recorded in a separate graph for each animal subject. Figure 3A–C shows the first part of therapy (first 15 minutes). Room temperature was ∼22°C. Before the therapy the deep tissues temperature was ∼35°C. After 5 minutes of the treatment the temperature of the skin increased to 40°C and the temperature of the adipose tissue increased to 42°C. The effect of the EFC system contributing the selective heating of the adipose tissue layer is noticeable after 10 minutes of therapy. Temperature difference between the skin and the adipose tissue layer was 4°C; skin temperature was 41°C, and the adipose tissue layer temperature is 45°C. Skin temperature remained on the tolerable level while the adipose tissue temperature reached the desired therapeutic level. The second group of graphs shows the second half of the therapy (next 15 minutes) (Fig. 4A–C). The broad electromagnetic field heated whole treatment area. The temperature of the adipose tissue layer increased approximately up to 44–45°C.

The device is engineered to primarily focus on the adipose tissue layer with specific impedance. This principle leads to the adipose tissue layer heating-up faster than other tissue layers. Also blood-rich tissues layers such as skin and muscle are cooled down by the circulating blood much faster than the adipose tissue layer. Furthermore, due to no contact application of the treatment head, the skin can be additionally cooled by circulating air.

**Fig. 5:**
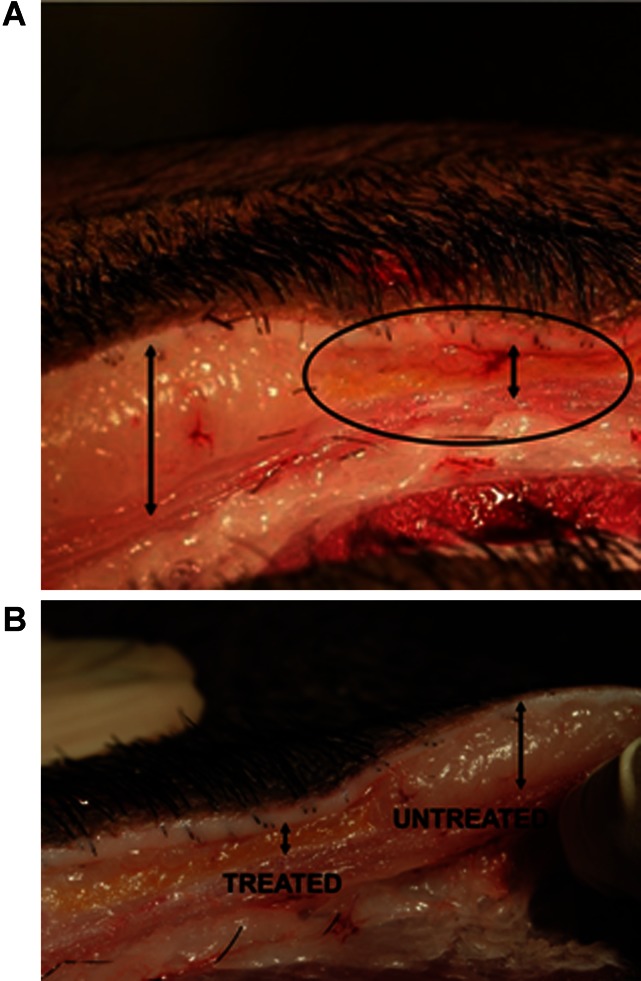
**A**,**B**: Local fat diminution on gross pathologic examination. A: Circle shows area in which energy was applied. Long arrow indicates no treatment. B: Treated area shows significant reduction of fat in subject 2.

Gross pathology examination revealed local skin depressions in all three animals. Skin section led through the lesion showed signs of an apoptotic reaction causing reduction of the adipose tissue layer (Fig. 5A,B). No other macroscopic signs were observed during gross pathology examination.

**Fig. 6:**
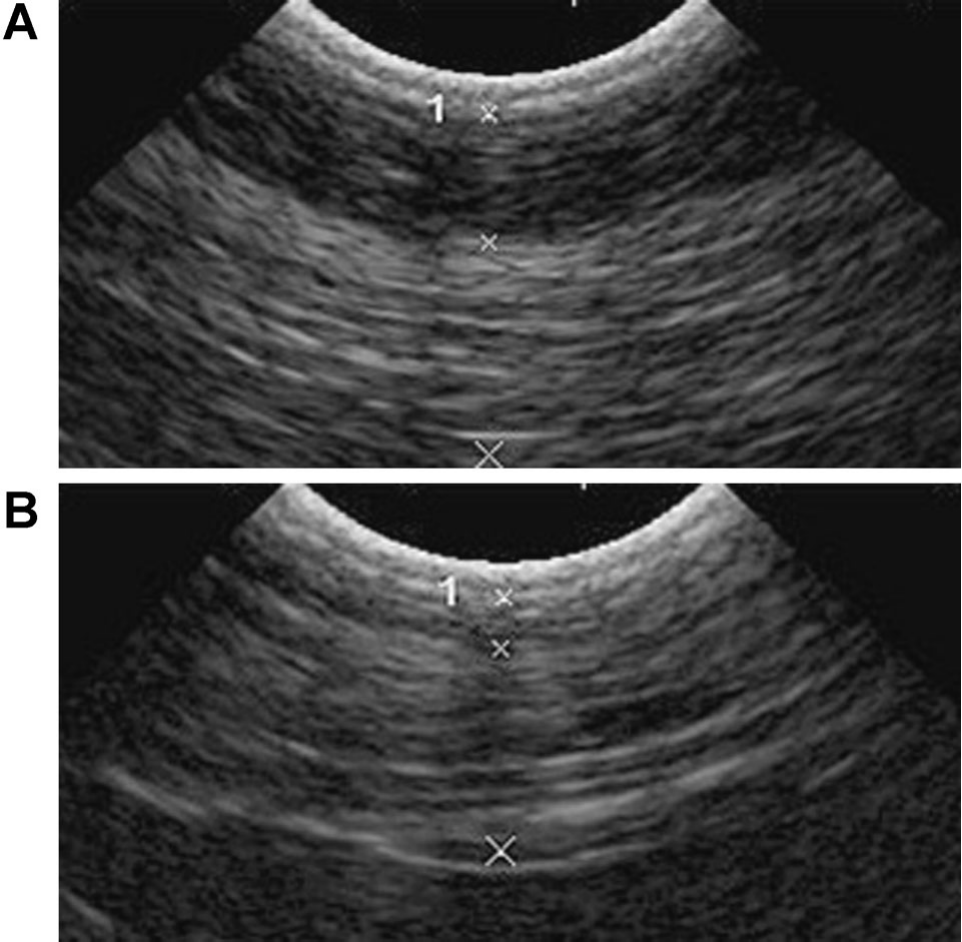
**A**: Before treatment fat layer is measured as 7.6 mm by Duplex ultrasound. **B**: After treatment thickness of adipose layer is reduced to 2.9 mm. (Between Xs and numbered as 1.)

Duplex ultrasound examination was performed to evaluate thickness reduction of adipose tissue. Thickness of the adipose tissue layer measured among all animals at the specific body area varied between 11.3 and 25.5 mm before the treatment. Before the fourth treatment adipose tissue layer was examined and the thickness of the tissue varied between 7.9 and 9.6 mm. At 3 months post-treatment, treated area were examined and loss of subcutaneous fat tissue was observed. Average adipose layer thickness reduction by ultrasound was calculated at 6.9 mm (see Fig. 6).

## DISCUSSION

Pathological examination clearly demonstrates the fat layer reduction in the treated area. Microscopic photographs of histology and TUNEL staining for apoptosis show that the reduction was most likely caused by the apoptotic phenomenon. Thermocouple results show that the adipose tissue is gradually heated up to the temperature of ∼45–46°C, while the skin temperature reaches only 42°C. A final 70% reduction of the abdominal fat layer was seen in the treated areas. Duplex ultrasound revealed a reduction of fat layer from 7.6 to 2.9 mm. Histologic evaluation revealed that epidermis, dermis, and adnexal structures such as hair follicles were unaffected by the treatment, while adipocytes were significantly affected.

This non-contact high frequency broad RF field device proved to be safe and effective in subcutaneous fat reduction in this porcine animal model. Laboratory, histological, or gross pathological analyses did not indicate any safety risks or side effects. A new model of fat reduction using high frequency waves has been successfully achieved in a porcine model. This has very positive implications in the development of an operator independent, contact-free device for reduction of human adipose tissue in clinical practice.
